# Safety of PRRSV-2 MLV vaccines administrated via the intramuscular or intradermal route and evaluation of PRRSV transmission upon needle-free and needle delivery

**DOI:** 10.1038/s41598-021-02444-3

**Published:** 2021-11-29

**Authors:** Adthakorn Madapong, Kepalee Saeng-chuto, Angkana Tantituvanont, Dachrit Nilubol

**Affiliations:** 1 Department of Veterinary Microbiology, Faculty of Veterinary Science, Chulalongkorn University, Henry Dunant Road, Pathumwan, Bangkok, 10330 Thailand; 2 Department of Pharmaceutics and Industrial Pharmacy, Faculty of Pharmaceutical Sciences, Chulalongkorn University, Bangkok, 10330 Thailand

**Keywords:** Live attenuated vaccines, Vaccines, Viral infection

## Abstract

Two distinct experiments (Exp) were conducted to evaluate the shedding and efficacy of 2 modified live porcine reproductive and respiratory syndrome virus (PRRSV) type 2 vaccines (MLV) when administered intramuscularly (IM) or intradermally (ID) (Exp A), and the potential of PRRSV transmission using a needle-free device (Exp B). One-hundred fifty-four, 3-week-old castrated-male, pigs were procured from a PRRSV-free herd. In Exp A, 112 pigs were randomly allocated into 4 groups of 21 pigs including IM/Ingelvac MLV (G1), IM/Prime Pac (G2), ID/Prime Pac (G3), and non-vaccination (G4). Twenty-eight remaining pigs were served as non-vaccination, age-matched sentinel pigs. G1 was IM vaccinated once with Ingelvac PRRS MLV (Ing) (Boehringer Ingelheim, Germany). G2 and G3 were IM and ID vaccinated once with a different MLV, Prime Pac PRRS (PP) (MSD Animal Health, The Netherlands), respectively. Following vaccination, an antibody response, IFN-γ-SC, and IL-10 secretion in supernatants of stimulated PBMC were monitored. Sera, tonsils, nasal swabs, bronchoalveolar lavage, urines, and feces were collected from 3 vaccinated pigs each week to 42 days post-vaccination (DPV) and assayed for the presence of PRRSV using virus isolation and qPCR. Age-matched sentinel pigs were used to evaluate the transmission of vaccine viruses and were introduced into vaccinated groups from 0 to 42 DPV. Seroconversion was monitored. In Exp B, 42 pigs were randomly allocated into 5 groups of 3 pigs each including IM/High (T1), ID/High (T2), IM/Low (T3), ID/Low (T4), and NoChal. Twenty-seven remaining pigs were left as non-challenge, age-matched sentinel pigs. The T1 and T2, and T3 and T4 groups were intranasally challenged at approximately 26 days of age with HP-PRRSV-2 at high (10^6^) and low (10^3^ TCID_50_/ml) doses, respectively. At 7 days post-challenge, at the time of the highest viremia levels of HP-PRRSV-2, T1 and T2, and T3 and T4 groups were IM and ID injected with Diluvac Forte using needles and a need-less device (IDAL 3G, MSD Animal Health, The Netherlands), respectively. Same needles or needle-less devices were used to inject the same volume of Diluvac Forte into sentinel pigs. Seroconversion of sentinels was evaluated. The results demonstrated that PP vaccinated groups (G2 and G3), regardless of the route of vaccination, had ELISA response significantly lower than G1 at 7 and 14 DPV. PP-vaccinated groups (G2 and G3) had significantly higher IFN-γ-SC and lower IL-10 secretion compared to the Ing-vaccinated group (G1). The two different MLV when administered intramuscularly demonstrated the difference in virus distribution and shedding patterns. PP-vaccinated pigs had significantly shortened viremia than the Ing-vaccinated pigs. However, ID-vaccinated pigs had lower virus distribution in organs and body fluids without virus shedding to sentinel pigs. In Exp B, regardless of the challenge dose, sentinel pigs intradermally injected with the same needle-less device used to inject challenged pigs displayed no seroconversion. In contrast, sentinel pigs intramuscularly injected with the same needle used to inject challenged pigs displayed seroconversion. The results demonstrated the transmission of PRRSV by using a needle, but not by using a needle-less device. In conclusion, our results demonstrated that ID vaccination might represent an alternative to improve vaccine efficacy and safety, and may be able to reduce the shedding of vaccine viruses and reduce the iatrogenic transfer of pathogens between animals with shared needles.

## Introduction

Porcine reproductive and respiratory syndrome (PRRS) is an economically devastating disease in pigs characterized by respiratory distress in finishing pigs and reproductive disorders in breeding dams^1,2^. PRRS virus (PRRSV), an enveloped, positive-sense, single-stranded RNA virus belonging to the genus Betaarterivius, subfamily Variarterivirinae family Arteriviridae within the order Nidoviralase is the causative agent^3^. PRRSV has been classified into two genetically distinct species including Betaarterivirus suid 1 (former PRRSV-1, European type) and Betaarterivirus suid 2 (former PRRSV-2, American type)^4–6^. PRRSV-1 has predominantly spread within European countries and currently has further evolved into 4 subtypes^7^. Meanwhile, PRRSV-2 has been dominant in the North American continent^8^ and has further evolved into 9 distinct lineages^9^. However, the co-existence of both PRRSV species has been reports in several Asian countries including Korea, Vietnam, and Thailand^10–12^. Additionally, variants of PRRSV-2 endemically present in Asia are genetically related to the highly pathogenic PRRSV-2 (HP-PRRSV-2) sublineage 8.7/HP-PRRSV-2 which has been a predominant virus in the region^9,12,13^.

Since its first emergence in the late 1980’s, PRRSV continues to cause economic losses to swine production worldwide. To control PRRS, several types of vaccines including inactivated, modified live vaccine (MLV) and subunit vaccines have been implemented in swine production worldwide with varying degree of success^14–16^. Presently, modified-live vaccines (MLVs) have been used more than inactivated and subunit vaccines in Asian countries. MLVs, regardless of the genotypes of the vaccine virus, have been implemented regularly in swine farms to control PRRSV^17,18^. Several PRRSV MLV vaccines, both based on PRRSV-1 and PRRSV-2, have been licensed in various countries depending on circulating PRRSV genotypes. However, concerns regarding the safety of PRRSV MLV has been raised by some studies demonstrating the shedding and persistence of vaccine virus in vaccinated hosts, in turn causing detectable viremia and potential transmission of vaccine virus to naïve animals^19,20^. In addition, the vaccine virus can cross the placental barrier in pregnant sows and infect the developing fetuses^21^ resulting in the transmission to naïve newborn piglets during lactation^1^. Finally, vaccine virus has been demonstrated to have a potential to merge with field virus in a recombination event, generating potential new genetically distinct variants of PRRSV in the farm that may contribute to virulence and disease incidence^22^. To deal with these potential issues, it is necessary to choose a PRRSV MLV that potentially has the least level of shedding and persistence of vaccine virus.

Intramuscular administration using needles has been the main route of vaccination in pigs. However, risks associated with needles have been increased. Therefore, alternative routes of vaccine administration are urgently considered. Needle-free devices have been used in human medicine to deliver antigen into skin^23^. Antigen delivery using needle free devices can be performed intradermally or transdermally. Presently, vaccination using needle-free devices is commercially available in pigs and intradermal vaccination of pigs has been shown to be able to trigger adaptive and humoral responses, even in the absence of adjuvant^24–27^. Currently, several commercially available vaccines against swine-relevant pathogens [i.e., *Mycoplasma hyopneumoniae*^28,29^, PRRSV^25,27^, porcine circovirus 2^30^, and pseudorabies virus^31,32^] are licensed for the intradermal needle-free delivery route. Apart from animal welfare benefits, intradermal needle-free vaccination has the potential to eliminate accidental needle-stick injuries by farm workers, reduce iatrogenic disease transfer from shared needles and reduce injection site lesions caused by intramuscular vaccination, which are known to be prevalent in swine at the slaughterhouse^33^. Additionally, it is also known that PRRSV can be transmitted through iatrogenic transfer between pigs when needles are reused during routine vaccination of PRRSV^34^.

Different PRRSV-2 MLV vaccines including Ingelvac PRRS MLV (Boehringer Ingelheim, Germany) and Prime Pac PRRS (MSD Animal Health, The Netherlands) have been increasingly used in swine herds in Southeast Asian countries^18,35,36^. There is a variety of vaccine delivery routes and commercial vaccines, and there is also increased use of intradermal needle-free delivery devices to administer PRRSV-2 MLV to pigs. However, there are only few reports that compare the safety of different PRRSV-2 MLVs, as well as reports that study the ability of needle-free devices to reduce iatrogenic disease transfer via needles^27,31^. Gathering together, a safe PRRSV-2 MLV delivered through a needle-free jet injector could potentially thus reduce virus shedding and persistence in the environment, as well as iatrogenic transfer of wild type PRRSV between vaccinated pigs.

Therefore, the objectives of the present study were to investigate safety issues, in terms of viral persistence in tissues and vaccine virus shedding to sentinel pigs, of 2 commercially available PRRSV-2 MLVs administered intramuscularly and intradermally. The antibody response, interferon-γ-secreting cells (IFN-γ-SC), IL-10 secretion were additionally observed. Moreover, the potential transmission of PRRSV from infected pigs to naïve pigs through either a needless device or conventional needle was investigated.

## Materials and methods

### Ethical statement for experimental procedures

All animal procedures were conducted following the recommendations in the Guild for the Care and Use of Laboratory Animal of the National Research Council of Thailand according to protocols reviewed and approved by the Chulalongkorn University Animal Care and Use Committee under protocol number 2031015, animal use license number UI-00058-2558. All methods were performed in accordance with the relevant guidelines and regulations. The study is reported in accordance with the ARRIVE guidelines (https://arriveguidelines.org).

### PRRSV vaccines, vaccination, and viruses

PRRSV vaccines used for vaccination were two PRRSV-2 MLVs including Ingelvac PRRS MLV (Boehringer Ingelheim, Rhein, Germany) and Prime Pac PRRS MLV (MSD Animal Health, Boxmeer, The Netherlands). Prime Pac PRRS MLV is available in two different preparations for intramuscular (IM) or intradermal (ID) vaccination. Dosage and administration routes were applied following the manufacturer’s instructions. In brief, a 2 ml dose of Ingelvac PRRS MLV (batch no2451218A) and a 1 ml dose of Prime Pac PRRS (batch no. A065CE04) was used for IM vaccination, respectively. A 0.2 ml dose of Prime Pac PRRS (batch no. A065CE04) was used for ID vaccination. ID vaccination and injection were performed using IDAL 3G needle-free device (MSD Animal Health, Boxmeer, The Netherlands).

Homologous vaccine viruses and highly pathogenic (HP)-PRRSV-2 were used in the present study. Homologous vaccine viruses refer to vaccine strains that were used as recall antigens for in vitro stimulation assay in the measurement of IFN-γ-SC and IL-10 production, as performed in previously described methods^37^. To challenge pigs, Thai PRRSV-2 (HP-PRRSV-2) isolate FDT10US23, an HP-PRRSV-2 variant genetically classified in the sublineage 8.7/HP-PRRSV-2 based on international systematic classification, according to the previously described method^9^, was used as a virus inoculum at the fifth passage in MARC-145 cells. The ORF5 genome sequence is available in GenBank under accession number JN255836. This isolate was isolated from swine herds experiencing PRRS outbreaks in the western region of Thailand during 2010–2011^13^. Pathogenesis and challenge studies of the challenged isolate were demonstrated according to previous studies^12,13,18,27,38^.

### Experimental design

One hundred fifty-four, castrated-male, PRRSV-free pigs at three weeks of age were procured from a PRRSV-free herd. Upon arrival, sera were collected individually and assayed for the presence of viral RNA and PRRSV-specific antibodies using PCR and ELISA to confirm their negative status. In the present study, two separate experiments were conducted. In experiment A (Exp A), pigs were vaccinated once with PRRSV-2 MLV via intramuscular (IM) or intradermal (ID) routes. PRRSV-specific antibodies and cell-mediated immunity were evaluated. The presence of the vaccine virus in tissues and organs and the shedding pattern to sentinel pigs were determined. In experiment B (Exp B), pigs were intranasally challenged with HP-PRRSV-2. At 7 days post-challenge (DPC), at the highest level of viremia, challenged pigs were injected with Diluvac Forte, either by the IM or ID route, using a conventional needle or needle-free device, respectively. The same needle or needle-free device was used to inject Diluvac Forte into sentinel pigs. Seroconversion of the sentinel pigs was determined. This was performed in order to investigate the virus transmission from infected to naïve pigs when using needle or needle-free device. Pigs in each group were kept in separate rooms with separated air spaces and monitored daily for physical condition and clinical respiratory disease throughout the experiment.

### Experiment A

One hundred and twelve pigs were randomly allocated based on weight stratification into 4 groups of 21 pigs each including IM/Ingelvac MLV (G1), IM/Prime Pac (G2), ID/Prime Pac (G3), and NoVac (G4) as showed in Table 1. Twenty-eight remaining pigs were served as non-vaccination, age-matched sentinel pigs. The IM/Ingelvac MLV (G1) group was IM vaccinated once with a 2 ml dose of Ingelvac PRRS MLV (Boehringer Ingelheim, Rhein, Germany). The IM/Prime Pac (G2) and ID/Prime Pac (G3) groups were vaccinated once via IM and ID routes with a 1 ml and 0.2 ml dose of Prime Pac PRRS (MSD Animal Health, Boxmeer, The Netherlands), respectively. ID vaccination was performed using IDAL 3G needle-free vaccinator. The NoVac (G4) group was left non-vaccination and the remaining 38 pigs were served as age-matched sentinel pigs.

**Table 1:** Experimental design of experiment A (Exp A).

Treatment group	Number of pigs	Vaccination	Vaccines	Vaccine type	Dosage and route of administration	Manufacturers
IM/Inglevac MLV (G1)	21	Yes	Ingelvac PRRS MLV	PRRSV-2	2 ml, IM	Boehringer Ingelheim, Rhein, Germany
IM/Prime Pac (G2)	21	Yes	Prime Pac PRRS	PRRSV-2	1 ml, IM	MSD Animal Health, Boxmeer, The Netherlands
ID/Prime Pac (G3)	21	Yes	Prime Pac PRRS	PRRSV-2	0.2 ml, ID	MSD Animal Health, Boxmeer, The Netherlands
NoVac (G4)	21	No	–	–	–	–

Four treatment groups included 3 vaccinated groups and 1 non-vaccinated group.

Routes of vaccine administration were included either intramuscular (IM) or intradermal (ID) using an IDAL 3G.

Blood samples were collected at 0, 7, 14, 21, 28, 35, 42, 49, 56, and 63 days post-vaccination (DPV). Sera were assayed for PRRSV specific antibody and PRRSV RNA using ELISA and quantitative RT-qPCR (qPCR), respectively. Peripheral blood mononuclear cells (PBMC) were isolated and used for in vitro stimulation to measure IL-10 production using an ELISA kit and IFN-γ-SC using an ELISPOT assay. Three pigs from each group were necropsied on weekly basis starting from 7 to 42 DPV. Sera, nasal swabs, tonsils, lungs, bronchoalveolar lavages (BAL), and feces were collected and assayed for the presence of PRRSV using qPCR and virus isolation in cell culture. The shedding of vaccine virus to naïve animals was measured by placing age-matched sentinel pigs in contact with the vaccinated pigs at 1, 7, 14, 21, 28, 35, and 42 DPV, 1 sentinel pig/group at each day. Each batch of sentinel pig was comingled with vaccinated pigs for 3 consecutive weeks. Sera were collected at 0, and 21 days post-commingling. Commingled pigs were weekly measured PRRSV specific antibody and PRRSV RNA using ELISA and qPCR, respectively.

### Experiment B

Forty-two pigs were randomly allocated based on weight stratification into 5 groups with 3 pigs each including IM/High (T1), ID/High (T2), IM/Low (T3), ID/Low (T4), and NoChal (T5) as showed in Table 2. Twenty-seven remaining pigs were left as non-challenge, age-matched sentinel pigs. Two different dosages of HP-PRRSV-2 were used to inoculate pigs. The IM/High (T1) and ID/High (T2) groups were intranasally inoculated with 4 ml (2 ml/nostril) of HP-PRRSV-2 (FDT10US23 isolate, 10^6^ TCID_50_/ml). The IM/Low (T3) and ID/Low (T4) groups were intranasally inoculated with 4 ml of HP-PRRSV-2 at a lower dose (FDT10US23 isolate, 10^3^ TCID_50_/ml). The NoChal (T5) group was served as a control, and the remaining 17 pigs were used as age-matched sentinel pigs. Sera were collected at 0, 7, 14, 21, and 28 DPC and assayed for the presence of PRRSV-specific antibody and RNA using ELISA and qPCR, respectively.

**Table 2:** Experimental design of experiment B (Exp B).

Treatment group	Number of pigs	Challenge	Detail of PRRSV-2 inoculum	Dosage and challenge route	PRRSV isolate	Diluvac Forte injection
IM/High (T1)	3	Yes	High dose group for IM injection	4 ml/pig, at 10^6^ TCID_50_/ml	HP-PRRSV-2 (FDT10US23)	1 ml/pig, IM
ID/High (T2)	3	Yes	High dose group for ID injection	4 ml/pig, at 10^6^ TCID_50_/ml	HP-PRRSV-2 (FDT10US23)	0.2 ml/pig, ID
IM/Low (T3)	3	Yes	Low dose group for IM injection	4 ml/pig, at 10^3^ TCID_50_/ml	HP-PRRSV-2 (FDT10US23)	1 ml/pig, IM
ID/Low (T4)	3	Yes	Low dose group for ID injection	4 ml/pig, at 10^3^ TCID_50_/ml	HP-PRRSV-2 (FDT10US23)	0.2 ml/pig, ID
NoChal (T5)	3	No	Negative control	–	–	–

Five treatment groups included 4 challenged groups and 1 non-challenged group.

Pigs were intranasally inoculated with HP-PRRSV-2 at 0 days post-challenge (DPC).

Pigs in non-challenge (NoChal) served as the control.

At 7 DPC, the challenged pigs were injected with Diluvac Forte via either IM or ID routes with the same needle or needle-less device used for inoculating pigs.

ID injection was performed using an IDAL 3G needle-free device.

To mimic vaccine administration, Diluvac Forte, an adjuvant, was used instead of a vaccine. At 7 DPC, the IM/High (T1) and IM/Low (T3) groups were IM injected with 1 ml of Diluvac Forte (batch no G197A01, MDS Animal Health, Boxmeer, The Netherlands) using a conventional needle (G18, 1″). The ID/High (T2) and ID/Low (T4) groups were ID injected with 0.2 ml of Diluvac Forte using IDAL 3G needle-free vaccinator (MSD Animal Health, Boxmeer, The Netherlands). The same conventional needles or needle-free device were used to inject the same volume of Diluvac Forte (MSD Animal Health, Boxmeer, The Netherlands) to sentinel pigs with the same route of injection. The same device that was used to inject Diluvac Forte to one infected pig were used to administer Diluvac Forte into 2 sentinel pigs. The NoChal group was left as a negative control. Blood samples were collected from sentinel pigs at 0, 7, 14, 21 and 28 days post-injection (DPI). Injected sentinel pigs were weekly measured PRRSV-specific antibody and PRRSV RNA using ELISA and qPCR, respectively.

### Clinical evaluation

Clinical signs were monitored daily post-vaccination and post-challenge periods for two consecutive weeks by the same person at the same time. The severity of clinical respiratory disease for each pig was evaluated using a scoring system following stress induction as previously described criteria^39^: 0 = normal, 1 = mild dyspnea and/or tachypnea when stressed, 2 = mild dyspnea and/or tachypnea when at rest, 3 = moderate dyspnea and/or tachypnea when stressed, 4 = moderate dyspnea and/or tachypnea when at rest, 5 = severe dyspnea and/or tachypnea when stressed, and 6 = severe dyspnea and/or tachypnea when at rest.

### Clinical sample collection

Blood was collected from pigs in serum separation tubes (Monovette, Sarstedt, Numbrecht, Germany) and centrifuged at 2000 × g for 10 min at 20 °C. Sera were stored in 1 ml aliquots at − 80 °C until used. Nasal swabs, tonsils, and feces were collected using individually packaged sterile swabs which were placed in Dulbecco’s modified eagle’s medium (DMEM, Gibco, MA, USA) supplemented with 5 × antibiotics (100 × Antibiotic-antimycotics, Gibco, MA, USA). Tonsils and feces were weighed and mixed with DMEM medium (10% weight by volume). Then, samples were homogenized and centrifuged at 4000 × g for 10 min. Homogenates were filtered through 0.2 μm pore size filters, treated with 5 × antibiotics overnight at 4 °C, and kept at − 80 °C until used. Urines were collected from bladders (10 ml/pig), filtered through 0.2 μm pore size filters, and kept at − 80 °C until used. Bronchoalveolar lavage (BAL) was performed aseptically at necropsy as previously described^40^. In brief, 50 ml of lavage fluid consisting of DMEM with 5 × antibiotics was gently dispensed and aspirated several times into the lungs. The BAL was kept at -80 °C until used.

### Antibody detection

PRRSV-specific antibodies were measured using a commercial ELISA kit (IDEXX PRRS X3 Ab test, IDEXX Laboratories Inc., MA, USA). The assay was performed following the manufacturer’s recommendation. Sera were considered positive for PRRSV antibody if the S/P ratio was greater than 0.4.

### Peripheral blood mononuclear cells (PBMC) isolation

Peripheral blood mononuclear cells (PBMC) were isolated from heparinized blood using gradient density centrifugation (Lymphosep, Biowest, MO, USA) as previously described^26^. Isolated PMBC were counted by an inverted microscope, and concentrations were accessed in cRPMI-1640 [RPMI-1640 media supplemented with 10% fetal bovine serum (FBS), 2 mM L-glutamine, and 50 μg/ml of gentamycin]. The viability of isolated PBMC was determined by Trypan blue (Sigma-Aldrich, MO, USA) staining and more than 90% viability were used for in vitro stimulation for IL-10 production and enzyme-linked immunospot (ELISPOT) assay as described below.

### Quantification of porcine IL-10

Porcine IL-10 concentration in the supernatant of stimulated PBMC was quantified using a porcine ELISA IL-10 kit (R&D Systems, MN, USA) under the manufacturer’s instructions. In brief, 2 × 10^6^ PBMC were seeded into 96-well plates and cultured in vitro for 24 h with either homologous vaccine viruses at 0.01 multiplicity of infection (MOI), phytohemagglutinin (PHA, 10 μg/ml, Sigma-Aldrich, MO, USA), or MARC-145 cell lysate (mock suspension). In each pig, the levels of porcine IL-10 secretion were calculated by subtracting the value of the mock-stimulated well from the PRRSV-stimulated well. Subtracted values were compared between treatment groups.

### ELISPOT assay

The number of PRRSV-specific IFN-γ-SC were determined in stimulated PBMC using a commercial ELISPOT IFN-γ kit (ELISpot porcine IFN-γ, R&D Systems, MN, USA). The assay was performed in accordance with manufacturer’s instruction and a previously described method^18^. Briefly, 2 × 10^5^ PBMC/well were seeded into 96-well plates and stimulated with homologous vaccine viruses at 0.01 MOI for 24 h. Phytohemagglutinin (PHA, 10 μg/ml, Sigma-Aldrich, MO, USA) and cRPMI-1640 were used as positive and negative controls, respectively. The spots were counted by an automated ELISPOT Reader (AID ELISPOT Reader, AID GmBH, Germany), and the background values were subtracted from the respective count of the stimulated cells and the immune response was expressed as the number of IFN-γ-SC per 10^6^ PBMC.

### Pathological examination

Three pigs from each vaccinated group were necropsied at 7, 14, 21, 28, 35, and 42 DPV. Macroscopic or microscopic lung lesions associated with PRRSV-induced pneumonia were evaluated as previously described^39^. For macroscopic lung lesions, each lung lobe was assigned a number to represent the approximate percentage of the volume of the entire lung and the percentage of the volume from each lobe added to the entire lung score (range from 0 to 100% of the affected lung). Sections were collected from all lung lobes as previously described^39^. Lung tissues were fixed in 10% neutral buffered formalin for 7 days and routinely processed and embedded in paraffin in an automated tissue processor. Sections were cut at 5 μm and stained with hematoxylin and eosin (H&E). For microscopic lung lesions, the lung sections were examined in a blinded manner and given an estimated score of the severity of interstitial pneumonia. In brief, 0 = normal; 1 = mild interstitial pneumonia; 2 = moderate multifocal interstitial pneumonia; 3 = moderate diffuse interstitial pneumonia, and 4 = severe diffuse interstitial pneumonia. The mean values of the microscopic lung lesion score of each group were calculated.

### Virus isolation

Virus isolation was performed in MARC-145 cells and PAM as previously described^41^. In brief, 100 μl of the filtered clinical sample was incubated in 96-well plates of monolayers of MARC-145 cells and PAM for 60 min at 37 °C to facilitate adsorption, washed twice with DMEM supplemented with 3% FBS (Gibco, MA, USA). Then, the plates were incubated for 3 days at 37 °C in a humidified atmosphere containing 5% CO_2_. Media were removed and cells were fixed with a cold acetone-methanol solution for 10 min and then air-dried. The virus was detected in a monolayer by indirect microscopy using PRRSV-specific monoclonal antibody (mAb) SR-30 (RTI, South Dakota, USA).

### Quantification of PRRSV RNA and RT-PCR

Total RNA was extracted from clinical samples using NucleoSpin Virus (Macherey-Nagel, Duren, Germany) according to the manufacturer’s instruction. The RNA quality was measured using a NanoDrop spectrophotometer (Colibri spectrometer, Titertek Berthold, Pforzheim, Germany). Copy number of PRRSV RNA in serum was quantified using probed-based real-time PCR as previously described^27^. The reaction was carried out in QuantStudio 3 Real-time PCR machine (Thermo-Fisher Scientific, MA, USA). To detects the presence of virus in clinical samples, extracted RNA was converted into cDNA and used for PCR which was performed using GoTaq Green Master Mix (Promega, WI, USA). Primer specific for the ORF5 gene and detection conditions were followed as previously described^37^.

### Statistical analysis

Analysis of variance (ANOVA) was performed to determine if there were significant differences among groups for each day separately. If the *P* value for an ANOVA table was less than or equal to 0.05, the difference between treatment groups was evaluated using a multiple comparison test. All data were analyzed using IBM SPSS Statistic for Windows version 22.0 (IBM, Armonk, NY, USA) (https://www.ibm.com/software/analytics/spss/register/).

## Results

### Experiment A

#### Clinical signs

All vaccinated pigs displayed no clinical abnormalities following vaccination, regardless of the route of vaccination.

#### PRRSV-specific antibody response

**Figure 1:**
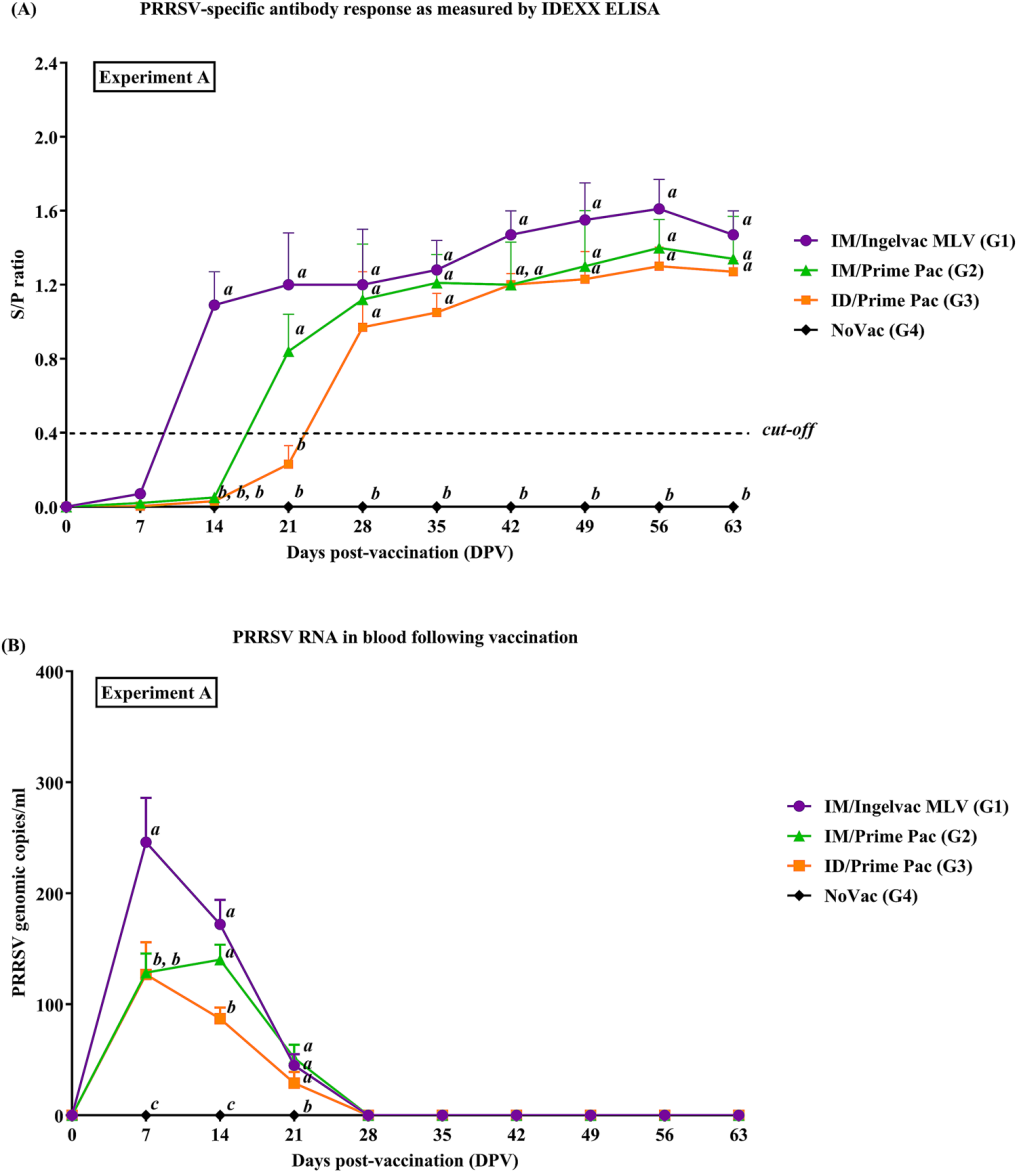
Mean values of (**A**) PRRSV-specific antibodies as measured by ELISA and (**B**) genomic copies of PRRSV RNA in the blood of pigs in Exp A. Values expressed as mean ± SEM. (**A**) Sample-to-positive (S/P) ratios equal to or greater than 0.4 were considered positive (cut-off value, dashed-line). The results were compared using two-way ANOVA for multiple comparisons. Different lowercase letters (*a*–*c*) indicate significant differences between treatment groups (*P* < 0.05) for each time-point.

The IM/Ingelvac MLV (G1) group had a detectable level of antibody response (0.12 ± 0.07) as measured by ELISA at 7 DPV but the level was lower than the cut-off level (S/P ratio < 0.4) (Fig. 1A). Antibody titers increased at 14 DPV and remained constantly at high levels until the end of the experiment. In contrast, the IM/Prime Pac (G2) and ID/Prime Pac (G3) groups had no detectable levels of PRRSV-specific antibody response at 7 and 14 DPV. At 14 DPV, the IM/Ingelvac MLV (G1) group had significantly higher PRRSV specific antibody titers than the IM/Prime Pac (G2) and ID/Prime Pac (G3) groups. At 21 DPV, increased antibody titers were detected in the IM/Prime Pac (G2) and ID/Prime Pac (G3) groups, but the ID/Prime Pac (G3) group had antibody titers below the cut-off level. The PRRSV specific antibody titer of the ID/Prime Pac (G3) group increased from 21 DPV and reached values above the cut-off level at 28 DPV. Antibody responses in the IM/Ingelvac MLV (G1) and IM/Prime Pac (G2) groups were significantly higher than that of the ID/Prime Pac (G3) group at 21 DPV. There was no difference in the antibody titers between 35 and 63 DPV.

#### Viremia

In all vaccinated groups, PRRSV RNA was detected and reached peaks at 7 DPV and gradually decreased to undetectable levels from 14 to 28 DPV (Fig. 1B). All vaccinated groups had no detectable levels of PRRSV RNA from 28 to 63 DPV. At 7 DPV, the IM/Ingelvac MLV (G1) group a had significantly higher (*P* < 0.05) PRRSV RNA level (246 ± 40.30 copies/ml) than that of the IM/Prime Pac (G2) and ID/Prime Pac (G3) groups. There was no difference between Prime Pac vaccinated groups, regardless to the vaccination routes. At 14 DPV, the ID/Prime Pac (G3) group had the lowest PRRSV RNA level (29.32 ± 12.13 copies/ml) compared to the other two groups and the difference was statistically significance. At 14 DPV, RNA levels in IM/Ingelvac MLV (G1) and IM/Prime Pac (G2) groups were not different.

#### Porcine IL-10

**Figure 2:**
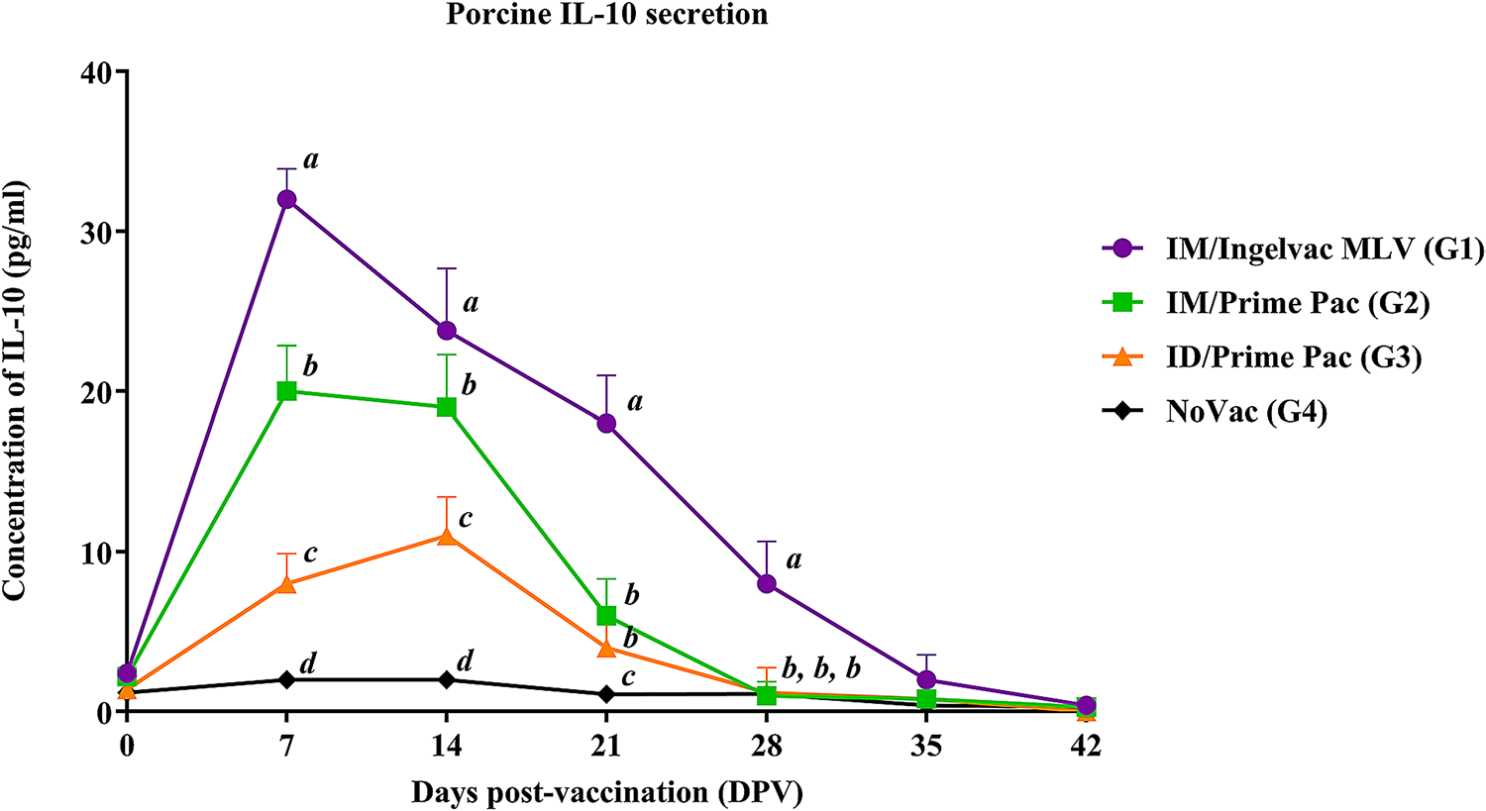
Quantification of porcine IL-10 secretion in stimulated PBMC with homologous viruses (vaccine viruses) of pigs in Exp A. Values expressed as mean ± SEM. The results were compared using two-way ANOVA for multiple comparisons. Different lowercase letters (*a*–*d*) indicate significant differences between treatment groups (*P* < 0.05) for each time-point.

Increased IL-10 levels were first detected in all vaccinated groups at 7 DPV, regardless of vaccination routes (Fig. 2). The IM/Ingelvac MLV (G1) group had the significantly highest IL-10 level (*P* < 0.05) than that of other vaccinated groups (range from 8.0 ± 1.5 to 32.0 ± 1.9 pg/ml) from 7 to 28 DPV. Meanwhile, the ID/Prime Pac (G3) group had the significantly lowest IL-10 level, ranging from 0.8 ± 1.2 to 11.0 ± 1.8 pg/ml, than that of other groups at 7 to 14 DPV. There was no difference in IL-10 levels between the IM/Prime Pac (G2) and ID/Prime Pac (G3) groups from 28 to 42 DPV.

#### PRRSV-specific IFN-γ-SC

**Figure 3:**
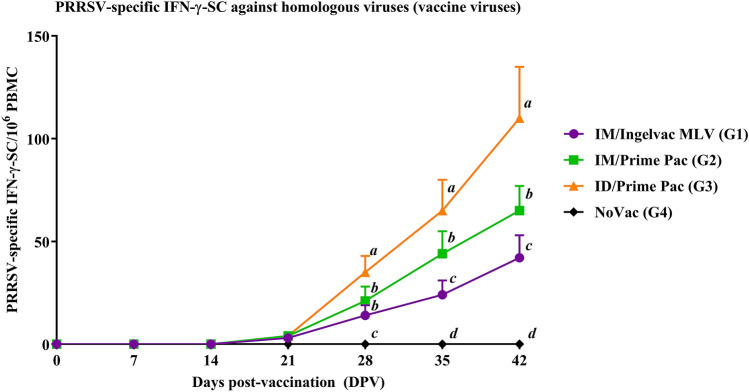
Frequencies of IFN-γ-SC after stimulation with homologous viruses (vaccine viruses) of pig PBMC in Exp A. Values expressed as mean ± SEM. The results were compared using two-way ANOVA for multiple comparisons. Different lowercase letters (*a*–*d*) indicate significant differences between treatment groups (*P* < 0.05) for each time-point.

PRRSV-specific IFN-γ-SC were first detected at 21 DPV in all vaccinated groups, but the levels were low (Fig. 3 and Supplementary Fig. S1). PRRSV-specific IFN-γ-SC were then gradually increased until 42 DPV in all vaccinated groups, regardless of the vaccination route. Statistical differences were observed at 28, 35 and 42 DPV. The ID/Prime Pac (G3) group had significantly (*P* < 0.05) higher IFN-γ-SC than those in other vaccinated groups at 28, 35, and 42 DPV. The IM/Prime Pac (G2) and IM/Ingelvac MLV (G1) groups showed no difference in the level of PRRSV-specific IFN-γ-SC at 28 DPV. However, the IM/Prime Pac (G2) group (44 ± 11 and 65 ± 12 cells/10^6^ PBMC) had significantly (*P* < 0.05) higher PRRSV-specific IFN-γ-SC than those in the IM/Ingelvac MLV (G1) group (24 ± 7 and 42 ± 11 cells/10^6^ PBMC) at 35 and 42 DPV.

#### Macroscopic and microscopic lung lesions

**Figure 4:**
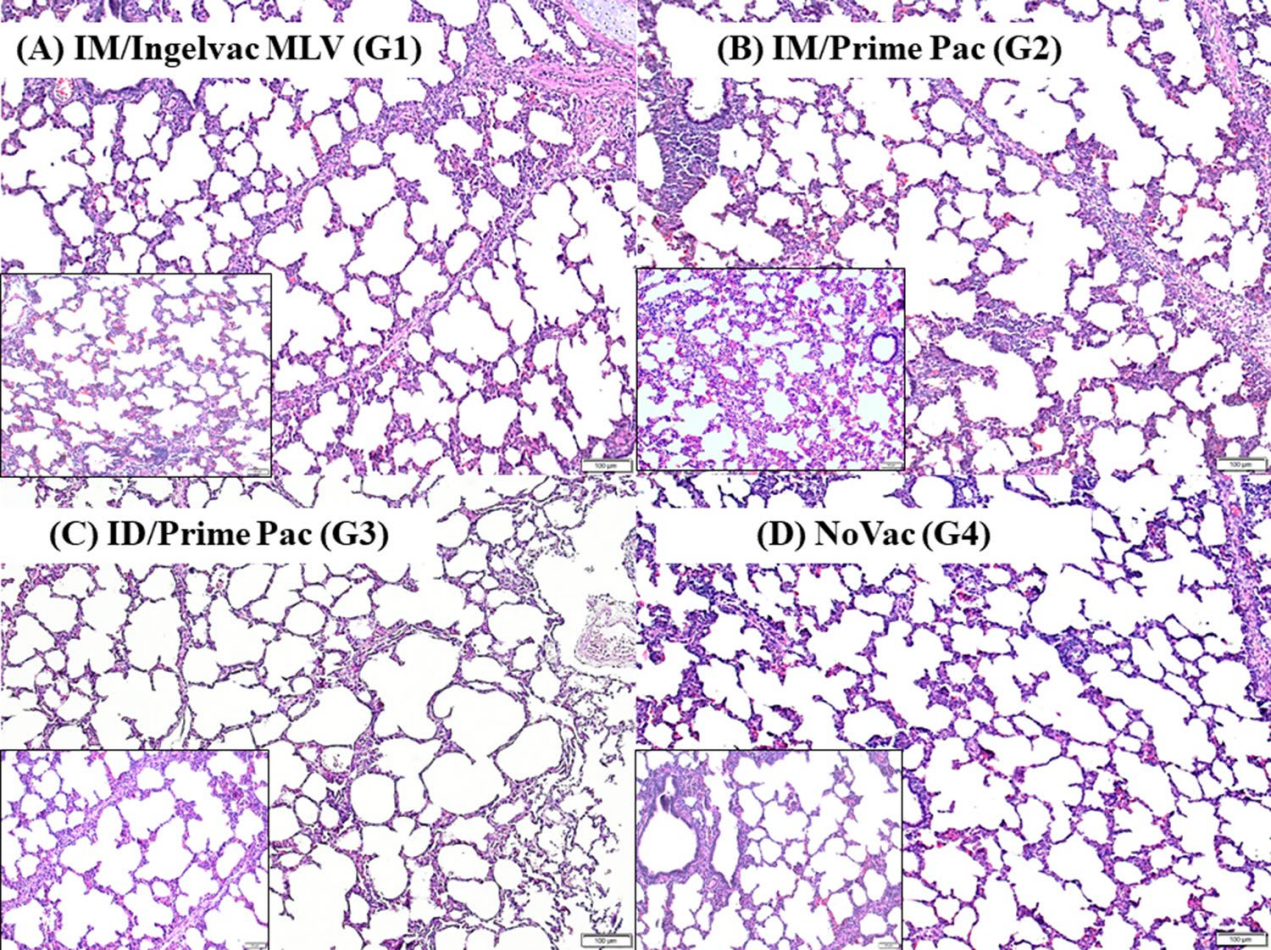
Microscopic lung lesion of pigs in Exp A; (**A**) IM/Ingelvac MLV (G1), (**B**) IM/Prime Pac (G2), (**C**) ID/Prime Pac (G3), and (**D**) NoVac (G4) groups, respectively. Black square pictures represented higher magnification (20X) of microscopic lung lesions for each group. H&E staining. Bars = 100 and 50 μm.

**Figure 5:**
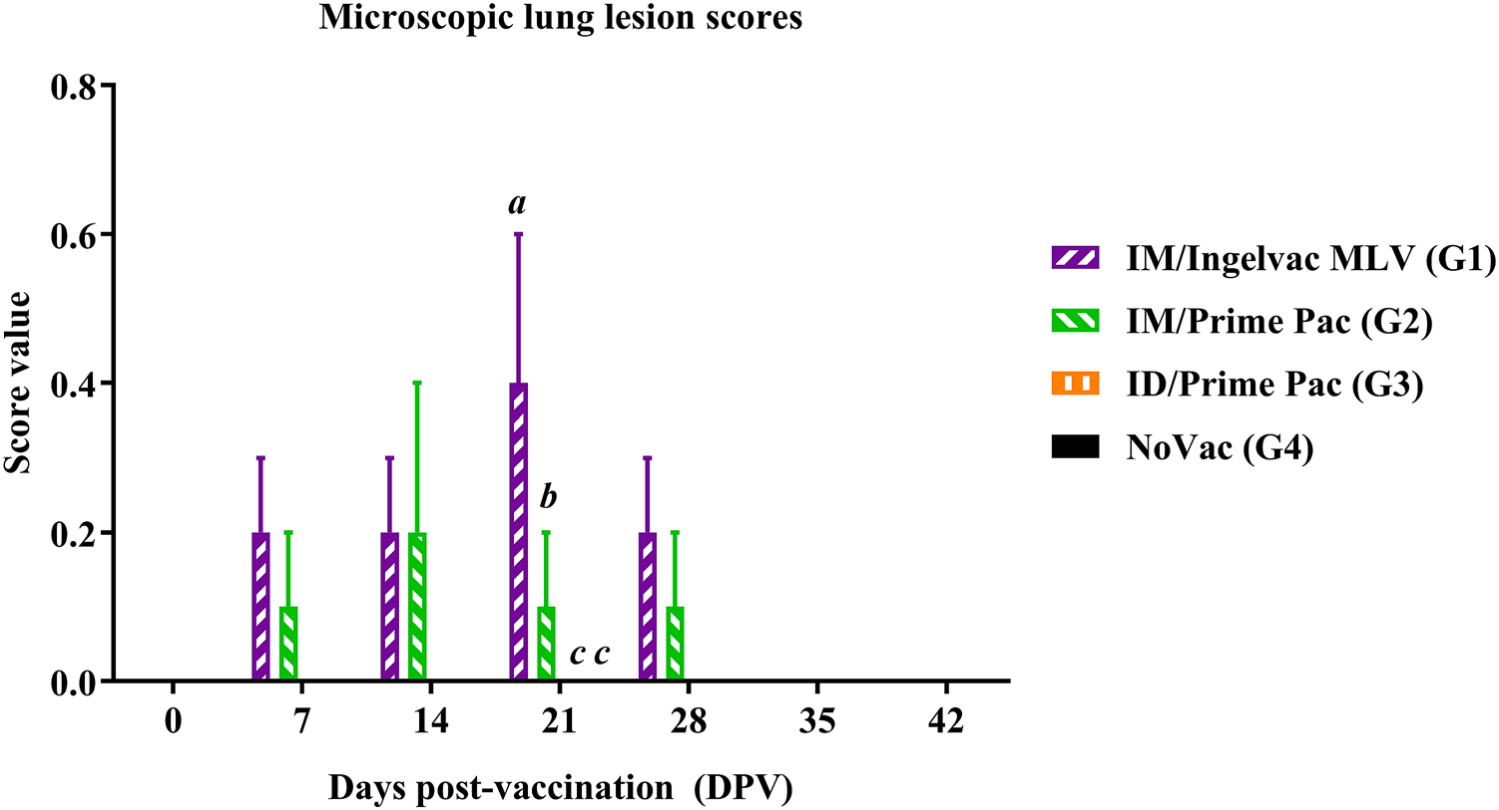
The mean values of the microscopic lung lesion scores of pigs in Exp A following vaccination. Values expressed as mean ± SEM. The results were compared using two-way ANOVA for multiple comparisons. Different lowercase letters (*a*–*c*) indicate significant differences between treatment groups (*P* < 0.05) for each time-point.

Macroscopic lung lesions were not detected in all vaccinated groups, regardless of the day of necropsy. For microscopic lung lesion score, the lesions were characterized by thickened septa with an increased number of interstitial macrophages and lymphocytes and by type II pneumocyte hyperplasia (Fig. 4). No microscopic lung lesions were detected in the ID/Prime Pac and NoVac groups, regardless of the necropsy day. The IM/Ingelvac MLV (G1) group had a significantly (*P* < 0.05) higher microscopic lung lesion score at 0.4 ± 0.2 than in the other vaccinated groups at 21 DPV (Fig. 5).

#### Detection of PRRSV in clinical samples by virus isolation

The results of virus isolation in MARC-145 cells and PAM cultures from different clinical samples collected at different necropsy days from treatment groups are summarized in Table 3. The frequency of PRRSV isolation varied depending on the clinical sample analyzed. PRRSV was more frequently isolated from tonsils than from any other samples and was not isolated from the feces and urine in both MARC-145 and PAM cell cultures, regardless of the day of necropsy and vaccine used. Following vaccination, all clinical samples derived from the ID/Prime Pac (G3) and NoVac (G4) groups were negative for PRRSV isolation regardless of the day of necropsy. PRRSV was detected in clinical samples of the IM/Ingelvac vaccinated group up to 42 DPV. PRRSV was detected in sera of 3/3, 3/3, and 2/3 pigs of the IM/Ingelvac MLV (G1) group on 7, 14, and 21 DPV, respectively. Meanwhile, PRRSV was isolated from sera of pigs in the IM/Prime Pac (G2) group at 7 and 14 DPV with 1/3 and 2/3 pigs, respectively. For nasal swab samples, PRRSV was isolated from pigs in the IM/Ingelvac MLV (G1) group at 28, 35 and 42 DPV with 2/3, 2/3, and 1/3 pigs detected, respectively. In contrast, 1 of 3 pigs from the IM/Prime Pac (G2) group had PRRSV-positive nasal swabs at 28 DPV only. For tonsils, PRRSV was isolated from 3/3, 3/3, 2/3, and 2/3 pigs in the IM/Ingelvac MLV (G1) group at 21, 28, and 42 DPV, respectively. In contrast, 1/3, 2/3 and 1/3 pigs in the IM/Prime Pac (G2) group were positive for PRRSV isolation at 21, 28, and 35 DPV, respectively. For the BAL samples, 1/3 and 2/3 pigs in the IM/Ingelvac MLV (G1) group were positive for PRRSV isolation at 21 and 28 DPV, respectively. PRRSV was detected in the BAL of 1/3 pigs in the IM/Prime Pac (G2) group at 28 DPV only.

**Table 3:** PRRSV-positive samples of experiment A (Exp A) using virus isolation in cell culture and PCR.

Treatment group	Sample	7 DPV			14 DPV			21 DPV			28 DPV			35 DPV			42 DPV		
		*VI1*	*VI2*	*PCR*	*VI1*	*VI2*	*PCR*	*VI1*	*VI2*	*PCR*	*VI1*	*VI2*	*PCR*	*VI1*	*VI2*	*PCR*	*VI1*	*VI2*	*PCR*
IM/Ingelvac MLV (G1)	Serum	**3/3**	**3/3**	**3/3**	**3/3**	**3/3**	**3/3**	**2/3**	**2/3**	**2/3**	0/3	0/3	0/3	0/3	0/3	0/3	0/3	0/3	0/3
	Nasal swab	0/3	0/3	0/3	0/3	0/3	0/3	0/3	0/3	0/3	**2/3**	**2/3**	**2/3**	**2/3**	**2/3**	**2/3**	**1/3**	**1/3**	**1/3**
	Tonsil	0/3	0/3	0/3	0/3	0/3	0/3	**3/3**	**3/3**	**3/3**	**3/3**	**3/3**	**3/3**	**2/3**	**2/3**	**2/3**	**2/3**	**2/3**	**2/3**
	BAL	0/3	0/3	0/3	0/3	0/3	0/3	**1/3**	**1/3**	**1/3**	**2/3**	**2/3**	**2/3**	0/3	0/3	0/3	0/3	0/3	0/3
	Urine	0/3	0/3	0/3	0/3	0/3	0/3	0/3	0/3	0/3	0/3	0/3	0/3	0/3	0/3	**1/3**	0/3	0/3	0/3
	Feces	0/3	0/3	0/3	0/3	0/3	0/3	0/3	0/3	0/3	0/3	0/3	0/3	0/3	0/3	0/3	0/3	0/3	0/3
IM/Prime Pac (G2)	Serum	**1/3**	**1/3**	**1/3**	**2/3**	**2/3**	**2/3**	0/3	0/3	0/3	0/3	0/3	0/3	0/3	0/3	0/3	0/3	0/3	0/3
	Nasal swab	0/3	0/3	0/3	0/3	0/3	0/3	0/3	0/3	0/3	**1/3**	**1/3**	**1/3**	0/3	0/3	0/3	0/3	0/3	0/3
	Tonsil	0/3	0/3	0/3	0/3	0/3	0/3	**1/3**	**1/3**	**1/3**	**2/3**	**2/3**	**2/3**	**1/3**	**1/3**	**1/3**	0/3	0/3	0/3
	BAL	0/3	0/3	0/3	0/3	0/3	0/3	0/3	0/3	0/3	**1/3**	**1/3**	**1/3**	0/3	0/3	0/3	0/3	0/3	0/3
	Urine	0/3	0/3	0/3	0/3	0/3	0/3	0/3	0/3	0/3	0/3	0/3	0/3	0/3	0/3	0/3	0/3	0/3	0/3
	Feces	0/3	0/3	0/3	0/3	0/3	0/3	0/3	0/3	0/3	0/3	0/3	0/3	0/3	0/3	0/3	0/3	0/3	0/3
ID/Prime Pac (G3)	Serum	0/3	0/3	0/3	0/3	0/3	0/3	0/3	0/3	0/3	0/3	0/3	0/3	0/3	0/3	0/3	0/3	0/3	0/3
	Nasal swab	0/3	0/3	0/3	0/3	0/3	0/3	0/3	0/3	0/3	0/3	0/3	0/3	0/3	0/3	0/3	0/3	0/3	0/3
	Tonsil	0/3	0/3	0/3	0/3	0/3	0/3	0/3	0/3	0/3	0/3	0/3	0/3	0/3	0/3	0/3	0/3	0/3	0/3
	BAL	0/3	0/3	0/3	0/3	0/3	0/3	0/3	0/3	0/3	0/3	0/3	0/3	0/3	0/3	0/3	0/3	0/3	0/3
	Urine	0/3	0/3	0/3	0/3	0/3	0/3	0/3	0/3	0/3	0/3	0/3	0/3	0/3	0/3	0/3	0/3	0/3	0/3
	Feces	0/3	0/3	0/3	0/3	0/3	0/3	0/3	0/3	0/3	0/3	0/3	0/3	0/3	0/3	0/3	0/3	0/3	0/3
NoVac (G4)	Serum	0/3	0/3	0/3	0/3	0/3	0/3	0/3	0/3	0/3	0/3	0/3	0/3	0/3	0/3	0/3	0/3	0/3	0/3
	Nasal swab	0/3	0/3	0/3	0/3	0/3	0/3	0/3	0/3	0/3	0/3	0/3	0/3	0/3	0/3	0/3	0/3	0/3	0/3
	Tonsil	0/3	0/3	0/3	0/3	0/3	0/3	0/3	0/3	0/3	0/3	0/3	0/3	0/3	0/3	0/3	0/3	0/3	0/3
	BAL	0/3	0/3	0/3	0/3	0/3	0/3	0/3	0/3	0/3	0/3	0/3	0/3	0/3	0/3	0/3	0/3	0/3	0/3
	Urine	0/3	0/3	0/3	0/3	0/3	0/3	0/3	0/3	0/3	0/3	0/3	0/3	0/3	0/3	0/3	0/3	0/3	0/3
	Feces	0/3	0/3	0/3	0/3	0/3	0/3	0/3	0/3	0/3	0/3	0/3	0/3	0/3	0/3	0/3	0/3	0/3	0/3

Values expressed as the number of the positive samples/total samples.

Significant values are in [bold].

*BAL* bronchoalveolar lavage, *DPV* days post-vaccination, *IM* intramuscular, *ID* intradermal, *PAM* porcine alveolar macrophages, *VI1* virus isolation in MARC-145 cells, *VI2* virus isolation in PAM.

#### Detection of PRRSV in clinical samples by PCR

The results of PRRSV detection by PCR from different clinical samples collected at different necropsy days from the treatment groups are summarized in Table 3. Similar to the results of virus isolation in cell culture, PRRSV detection by PCR varied depending on the clinical samples of each necropsy day regardless of the vaccine used. There was no PRRSV RNA in feces of all the treatment groups. Clinical samples derived from the ID/Prime Pac (G3) and NoVac (G4) groups were negative for PRRSV detection. PRRSV RNA was detected in sera of 3/3, 3/3, and 2/3 pigs in the IM/Ingelvac MLV (G1) group at 7, 14 and 21 DPV, respectively. Meanwhile, sera of pigs in the IM/Prime Pac (G2) group were positive for PRRSV with 1/3 and 2/3 pigs at 7 and 14 DPV, respectively. Like PRRSV isolation results, nasal swab samples from pigs in the IM/Ingelvac MLV (G1) group were positive for PRRSV at 28, 35, and 42 DPV with 2/3, 2/3, and 1/3 pigs, respectively. Nasal swabs of pigs in the IM/Prime Pac (G2) group were positive for PRRSV at 28 DPV with 1/3 pigs detected. PRRSV was detected in the tonsils from pigs in the IM/Ingelvac MLV (G1) group at 21, 28, 35, and 42 DPV with 3/3, 3/3, 2/3, and 2/3 pigs detected, respectively. For the BAL, pigs in the IM/Ingelvac MLV (G1) group were positive for PRRSV at 21 and 28 DPV with 1/3 and 2/3 pigs detected, respectively. One out of 3 pigs in the IM/Prime Pac (G2) group had PRRSV positive in the BAL at 28 DPV only (Table 3). PRRSV was detected in the urine sample from a pig (1 out of 3) in the IM/Ingelvac MLV (G1) group at 35 DPV and the urine samples from the IM/Prime Pac (G2) group showed no PRRSV positive.

#### Seroconversion of sentinel pigs

None of the sentinel pigs introduced to the ID/Prime Pac (G3) and NoVac (G4) groups were seropositive. Sentinel pigs introduced to the IM/Ingelvac MLV (G1) group at 7, 14, 21, and 28 DPV seroconverted. In contrast, the sentinel pigs introduced to the IM/Prime Pac (G2) group were seropositive only at 21 DPV.

### Experiment B

#### Clinical signs

Pigs in the IM/High (T1) and ID/High (T2) groups displayed the clinical respiratory disease associated with PRRSV. Meanwhile, the IM/Low (T3) and ID/Low (T4) groups showed low or no clinical symptoms.

#### PRRSV specific antibody response

**Figure 6:**
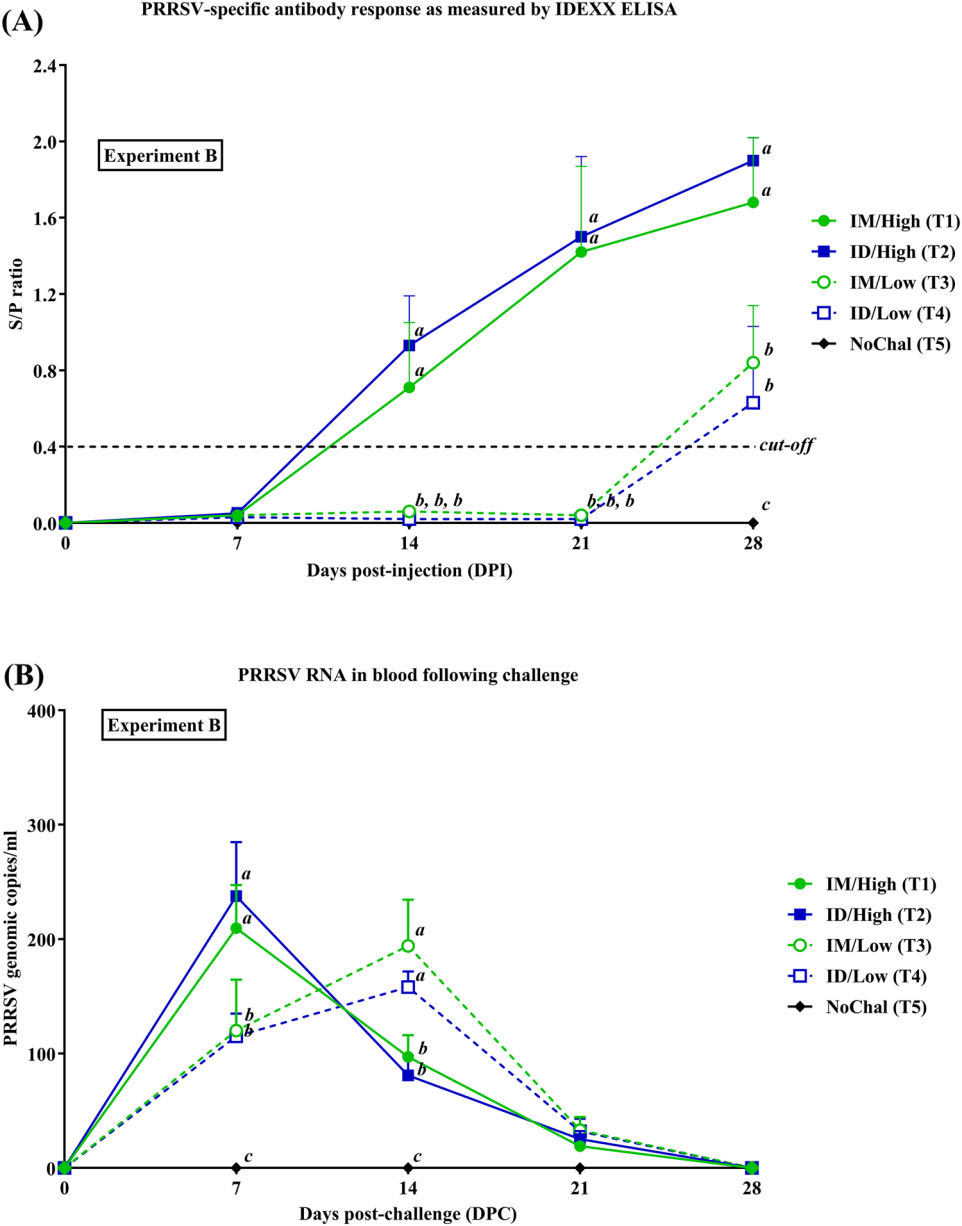
Mean values of (**A**) PRRSV-specific antibodies as measured by ELISA and (**B**) genomic copies of PRRSV RNA in the blood of pigs in Exp B. Values expressed as mean ± SEM. (**A**) Sample-to-positive (S/P) ratios equal to or greater than 0.4 was considered positive (cut-off value, dashed-line). The results were compared using two-way ANOVA for multiple comparisons. Different lowercase letters (*a*–*c*) indicate significant differences between treatment groups (*P* < 0.05) for each time-point.

The induction of the PRRSV-specific antibody response in challenged pigs in Exp B was dose-dependent (Fig. 6A). The IM/High (T1) and ID/High (T2) groups had a detectable antibody response at 14 DPC. Significantly increased antibody tiers were observed in both groups from 14 to 28 DPC. In contrast, PRRSV-specific antibody titers of the IM/Low (T3) and ID/Low (T4) groups were first detected at 28 DPC. The antibody levels of IM/High (T1) and ID/High (T2) groups were significantly higher than that of the IM/Low (T3) and ID/Low (T4) groups from 14 to 28 DPC (Fig. 6A).

#### Viremia

Following the challenge, PRRSV RNA in challenged groups with high doses rapidly increased and reached peaks at 7 DPC then gradually decreased to negativity from 14 to 28 DPC (Fig. 6B). In contrast, PRRSV RNA in challenged groups with low doses had continually increased and reached peaks at 14 DPC then rapidly decreased to negativity at 28 DPC. The IM/High (T1) and ID/High (T2) groups had significantly higher (*P* < 0.05) PRRSV RNA (range from 209.51 ± 37.7 to 237.60 ± 47.17 copies/ml) than that of the IM/Low (T3) and ID/Low (T4) groups at 7 DPC (range from 115.12 ± 20.22 to 120.53 ± 45.28 copies/ml). Meanwhile, PRRSV RNA in the IM/Low (T3) and ID/Low (T4) groups were significantly (*P* < 0.05) higher (range from 32.21 ± 4.21 to 194.43 ± 40.74 copies/ml) than that of the IM/High (T1) and ID/High (T2) groups (range from 19.09 ± 3.03 to 97.00 ± 19.14 copies/ml) at 14 DPC (Fig. 6B).

#### Seroconversion of sentinel pigs

None of the sentinel pigs injected with Diluvac Forte via ID route was seropositive (Table 4). In contrast, sentinel pigs injected via IM route had seroconversion from 14 to 28 DPI, regardless of doses of PRRSV challenge. At 14 DPI, 4 and 2 of 6 sentinel pigs of the IM/High (T1) and IM/Low (T3) groups were seropositive, respectively. All 6 sentinel pigs both IM/High (T1) and IM/Low (T3) groups were seropositive at 21 and 28 DPI.

**Table 4:** Seroconversion of sentinel pigs in experiment B (Exp B) as measured by ELISA.

Experiment B treatment group	Apparatus for Diluvac Forte injection	Seroconversion of sentinel pigs after injection with Diluvac Forte				
		Days post-injection (DPI)				
		0	7	14	21	28
IM/High (T1)	Conventional needle (G18, 1″)	0/6	0/6	**4/6**	**6/6**	**6/6**
ID/High (T2)	Needle-free (IDAL 3G)	0/6	0/6	0/6	0/6	0/6
IM/Low (T3)	Conventional needle (G18, 1″)	0/6	0/6	**2/6**	**6/6**	**6/6**
ID/Low (T4)	Needle-free (IDAL 3G)	0/6	0/6	0/6	0/6	0/6
NoChal (T5)	–	0/6	0/6	0/6	0/6	0/6

Sera were considered positive for PRRSV antibodies if the S/P ratio was greater than 0.4

Values expressed as the number of positive sentinel pig(s)/total number of sentinel pigs (n = 6).

Significant values are in [bold].

## Discussion

A conventional needle is a routine procedure for vaccine administration in swine industry in Southeast Asian countries. Many problems including broken needles in swine meat, muscle damage, abscesses, and infection and transmission by other pathogens, PRRSV, for instance, were found. In addition, a recent outbreak of highly infectious swine pathogen, African Swine Fever virus (ASFV) in Asia has raised a concern of transmission over a conventional needle. The use of a shared needle under this circumstance could be the risk of virus transmission not only PRRSV but also ASFV. The reduction of careless needle stick injuries to improve carcass quality and prevention of virus transmission are highly concern. In the present study, we would like to emphasize the safety and pathogen transmission during vaccine administration of intradermal vaccination using a needle-free device. PRRSV infection was used as a model. Therefore, the two studies were conducted to investigate the safety of vaccination with two PRRSV-2 MLV, either by IM or ID routes, in terms of the immune response, vaccine virus distribution in tissues and organs, and shedding patterns to sentinel pigs. The transmission of virus through a needless device and conventional needle were additionally investigated. Following vaccination, it was demonstrated that different PRRSV-2 MLV had different outcomes of the induction of an immune response and safety. It was notable that there were differences in early antibody detection between two PRRSV-2 MLV. ID Prime Pac PRRS vaccinated pigs showed delayed antibody response compared to that of Ingelvac PRRS MLV vaccinated pigs and IM Prime Pac vaccinated pigs. However, an ELISA response has long been recognized for its un-relationship with protection, unlike a response measured by viral neutralization assay. However, major findings in the present study are the induction of IFN-γ-SC and IL-10. The IFN-γ-SC in the ID Prime Pac vaccinated group had significantly higher frequencies than the other two IM vaccinated groups following vaccination. Increased IL-10 production was observed in all vaccinated groups following vaccination, and the ID vaccinated group had significantly lowest IL-10 levels compared to that of both IM vaccinated groups. For the evaluation of the safety following vaccination, our findings showed that Prime Pac vaccinated pigs had lower levels of viremia, decreased the frequency of PRRSV distribution in organs, and shortened virus shedding compared to that of the Ingelvac PRRS MLV vaccinated pigs. Interestingly, an ID vaccination was best able to shorten the viremic phase, reduced the frequency of PRRSV distribution in organs, and shorten virus shedding compared to that of the intramuscular route. Our findings demonstrate that PRRSV distribution was found in intramuscular vaccinated groups but not in the intradermal vaccinated group. In addition, the use of conventional needles led to biological and mechanical transmissions of PRRSV between vaccinated or infected pigs, and naïve pigs but the transmission was not observed in the use of a needle-free device. ID vaccination might represent an alternative to improve vaccine efficacy and safety, and may be able to reduce the shedding of vaccine viruses and reduce the iatrogenic transfer of pathogens between animals with shared needles.

For the humoral immune response, the results of the present study demonstrated that the induction of the humoral immune response of PRRSV MLV was different, regardless of vaccination routes. In agreement with our previous study, antibody response against PRRSV following vaccination with PP was delayed and reached values above the cut-off levels at 21 DPV^37^. The differences in the induction of PRRSV specific antibody response as measured by ELISA between IM and ID vaccinated pigs are in accordance with findings from previous studies^26,27^. The mechanisms responsible for the induction of humoral immune response were not fully understood but the specific virus isolate used for the vaccines might play an important role.

Regarding cell-mediated immune response, delivery of antigen through the intradermal route could induce T cell polarization via the Th1 pathway, favoring the induction of IFN-γ which was observed in the present study. The ID-vaccinated pigs showed a significantly higher IFN-γ-SC response than that of the IM-vaccinated pigs. These findings are in agreement with previous reports in which the ID-vaccinated pigs induce relatively more IFN-γ-SC than that of the IM-vaccinated pigs^25–27^. One possible factor associated with this finding is the presence of skin-resident immune cells capable of sufficiently capture antigens directly from the skin. The skin is rich in professional antigen-presenting cells (APC), including epidermal Langerhans cells (LC) and dermal dendritic cells, which are known to relocate to draining lymph nodes and trigger immune responses^42^.

Another possibility of higher IFN-γ-SC in the ID-vaccinated group could be due to the lower IL-10 production. Our results showed that the IL-10 concentration was lower in ID-vaccinated pigs than in the IM-vaccinated pigs following vaccination. These findings are in agreement with previous reports in which both ID- and IM-vaccination induce IL-10 production, but the ID-vaccinated pigs induced significantly lower IL-10 levels than that of the IM-vaccinated pigs^26,27^. Nevertheless, the delivery of antigen through the intradermal route could target dendritic cells. IL-10 is a cytokine of the Th2 response. The delivery through this route could induce T cell polarization through the Th1 pathway, favoring other cytokines that act against Th2 cells^43^. The differences in IL-10 production among the PRRSV MLV vaccination group are not surprising. Previous reports demonstrated that PRRSV isolates vary in the degree of IL-10 production both in vivo and *in vitro*^44,45^. Additionally, the induction of IL-10 might depend on the virus isolate used in the experiment and the vaccine^46–48^. However, the mechanism of IL-10 induction following vaccination by IM and ID routes are not understood.

The results of the present study demonstrated that some differences can be established in the safety of the 2 PRRSV MLV compared, although not all parameters were evaluated. Consequently, no differences in the induction of clinical signs were observed among vaccinated groups. Also, no clinical signs were recorded for any pig throughout the experiment. Although the macroscopic lung lesions can be considered similar between vaccinated groups, the microscopic lung lesions increased over time until 28 DPV and resolved at 35 DPV. This finding is in accordance with previous reports in which indicate that lung lesions generated by PRRSV-2 MLV isolates tend to be more persistent and last longer than those produced by PRRSV-1 MLV isolates^20,49^. Therefore, it is assuring that the patterns of lung lesions observed in the present study are more relevant to the virus isolate used for the vaccine than the changes occurring through the attenuation process^20^. Nonetheless, since the pathophysiological characteristics of virus isolates used for the vaccine could not be determined in the present study, the impact of the attenuation process in the occurrence and progression of lung lesions cannot completely ruled out.

Our results showed that the microscopic lung lesions of pigs vaccinated with Ingelvac PRRS MLV were significantly higher compared to PP vaccinated pigs on 21 DPV. These findings might be due to the difference in the intrinsic characteristics of the parent strains and, in particular, in their pneumotropism and pathological effects in the host might be related to these differences, that have been previously described^39,50^. Contrarily, the attenuation process might have altered the tropism of the vaccine viruses and restricted their ability to replicate in the lungs, and cause lung pathology. Remarkably, the frequency of virus isolation from the lung obtained in the present study was relatively lower than that of the other studies carried out with wide-type viruses^49,51^, indicating that the attenuation process of the vaccine virus might affect the cell tropism in the lung.

In addition to their ability to induce an immune response, the shedding patterns of PRRSV-2 MLV were evaluated using three different measurements, including the duration of viremia, the distribution of vaccine virus in organs, and the infection in sentinel pigs. Following vaccination, differences in the magnitude of viremia and the percentages of PRRSV-positive samples between PRRSV-2 MLV vaccinated groups were observed. Although the dynamics of viremia were similar among vaccinated groups, the magnitude of viremia of pigs vaccinated with Ingelvac PRRS MLV was statistically higher than those of the PP vaccinated pigs. In the same way, the percentage of PRRSV-positive tissue samples was higher in pigs vaccinated with Ingelvac PRRS MLV than in pigs vaccinated with the PP. These findings suggest that the viremic phase and the vaccine virus distribution in tissues were associated with the virus isolates use as vaccines^37^. However, the virus distribution in organs and the shedding patterns of the vaccine virus over the long period of time are limited, the shedding of vaccine virus could be occurred beyond our desired time point. Notably, the ID-vaccinated pigs show no PRRSV-positive samples regardless of the detection analyses, although the magnitude of viremia of the ID-vaccinated pigs was similar to the IM-vaccinated pigs. The discrepancy between viremia and virus isolation could be due to the fact that the ability to replicate in the target cells of the vaccine virus is restricted or that the quantity of virus in serum was lower than the limit of detection of the qPCR. To postulate, additional studies are needed.

An HP-PRRSV-2 isolate was used as a challenge virus because it has been a predominant virus in Asian countries^12^, and its virulence was well recognized. The results generated herein could be valuable information to swine farms in this region. The HP-PRRSV-2 strain used for the challenge in the present study was grouped in the sublineage 8.7/HP-PRRSV-2. Although the HP-PRRSV isolate has been well studied, in terms of pathogenesis and virulence, virus shedding and transmission into other pigs or environment have not been characterized yet. The transmission of virus to other pigs infected with genetically distinct PRRSV isolates could potentially results in recombination. The recombination between field PRRSV strains could resulted in the greater PRRSV pathogenicity. The recombination between HP-PRRSV-2 and the NADC30 isolate that has been confirmed as highly pathogenic^52^ or an HP-PRRSV-2 vaccine-like strain circulating in the field followed by PRRSV vaccination has been reported^53^. According to recent findings^22^, recombination events may occur between MLV and field strains of the virus. The reduction of MLV shedding post-vaccination may be able to reduce the potential of this recombination event occurring. Several other studies have also documented known vaccine strains recombining with field strains to form more pathogenic strains^54^. PRRSV is known to have a high degree of survival in the environment^55^. Working on the assumption that survival is similar to that of wild-type isolates, vaccine viruses shed into the environment may become longer, be re-ingested by swine and play a part in new recombination isolates arising on the farm. However, we acknowledge that type of PRRSV MLV may have been attenuated through cell culture passage and hence have a decreased level of survival in the environment. Additional studies to investigate the survival of the vaccine virus in the environment and the duration of vaccine virus shedding are needed. Besides, our results suggest that the lower the vaccine virus shedding of the PRRSV MLV vaccine, the lesser the recombination events between the vaccine strain and wild-type isolates.

Regarding the immunological and virological outcomes of the HP-PRRSV-2 challenge, it was evident that the high dose challenged pigs developed rapidly viremia and a PRRSV-specific antibody response compared to the low dose challenged pigs. These finding demonstrates that the onset of the immune response and viremia was associated with challenge dose^56^. Supporting the case for intradermal needle-free vaccination, the Exp B illustrates the cessation of iatrogenic pathogen transfer between swine using a jet injector needle-free device (IDAL 3G), using PRRSV as a model in swine vaccinated with shared needles. Jet injection devices have been trialed in human medicine and utilize a high-pressure fluid jet, propelled by a spring or gases, to breach the skin, before introducing the vaccine antigen through a low-pressure jet^57^. Theoretically, there is no possibility of pathogens being transferred from animal to animal using this device. However, it has been documented in the past that certain devices can transfer pathogens due to liquid splashing back into the device as the low-pressure stream ceases^58,59^. It is likely that differences exist across devices due to different design mechanics and that not all devices are hence equal. In conventional swine farming, needles are often shared between pigs that are being vaccinated. Needles shared in such a manner have been documented in human medicine to be capable of transmitting pathogens such as HIV^60^, and it is likely that for swine it is no different. Unfortunately, this is common practice on commercial swine operations due to the need for worker speed and efficiency. It is important to note that this study only assessed the potential for the vaccination ‘jet’ to transmit diseases and that great care was taken not to contaminate the device exterior between animals with fecal matter or other secretions. Given the highly transmissible nature of PRRSV in vivo^34^, it could be imagined that in a conventional situation, extraneous contamination of the devices may cause transmission of PRRSV via other routes, such as the oral-fecal route.

## Conclusions

The present studies illustrated that different PRRSV-2 MLV had similar patterns of the induction of antibody response, but the differences were observed in the early phase following vaccination. The intradermal vaccination might represent an alternative to improve vaccine safety, as it induced lower IL-10 levels and more IFN-γ-SC as well as the reduction of virus shedding within the herd and reduce the iatrogenic transfer of pathogens between animals with shared needles.

## Supplementary Information

Supplementary Figure S1.
