# Supplementary material for: Safety of PRRSV-2 MLV vaccines administrated via the intramuscular or intradermal route and evaluation of PRRSV transmission upon needle-free and needle delivery
Source: Sci Rep. 2021 Nov 29;11:23107. doi: 10.1038/s41598-021-02444-3 (PMC8629989; doi:10.1038/s41598-021-02444-3)
Supplement: Supplementary file 1 — Supplementary Figure S1. [file 41598_2021_2444_MOESM1_ESM.pdf]

Supplementary Information

Safety of PRRSV-2 MLV vaccines administrated via the intramuscular or intradermal route and evaluation of PRRSV transmission upon needle-free and needle delivery

Adthakorn Madapong, Kepalee Saeng-chuto, Angkana Tantituvanont and Dachrit Nilubol

Supplementary Figure

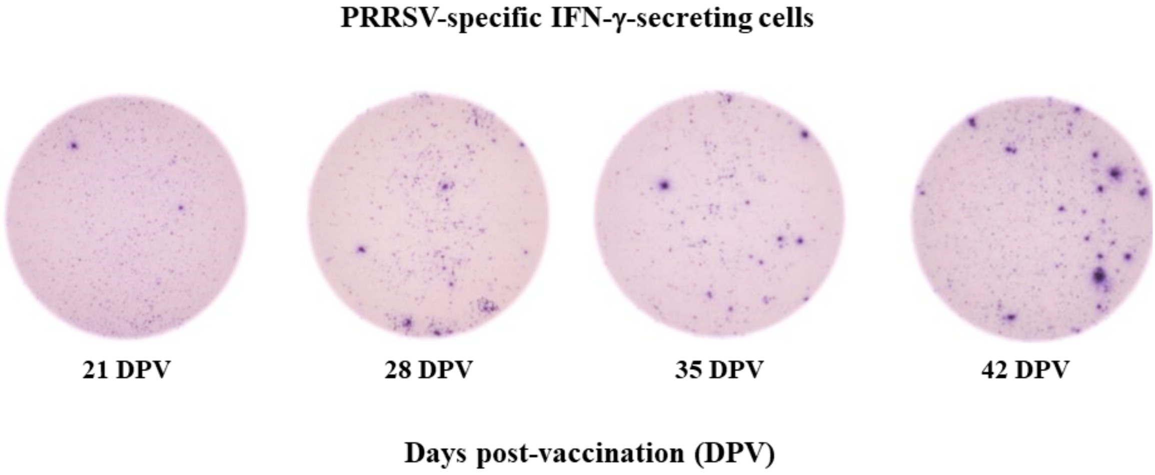

Supplementary Figure S1. The representative of PRRSV-specific IFN-γ-SC ELISPOT in stimulated PBMC with homologous vaccine virus at 0.01 multiplicity of infection (MOI) of pigs in Exp A.
